# Evaluating and benchmarking the EEG signal quality of high-density, dry MXene-based electrode arrays against gelled Ag/AgCl electrodes

**DOI:** 10.1088/1741-2552/ad141e

**Published:** 2024-01-12

**Authors:** Brian Erickson, Ryan Rich, Sneha Shankar, Brian Kim, Nicolette Driscoll, Georgios Mentzelopoulos, Guadalupe Fernandez-Nuñez, Flavia Vitale, John D Medaglia

**Affiliations:** 1 Applied Cognitive and Brain Sciences, Department of Psychology, Drexel University, Philadelphia, PA 19104, United States of America; 2 Department of Bioengineering, University of Pennsylvania, Philadelphia, PA 19104, United States of America; 3 Center for Neuroengineering and Therapeutics, University of Pennsylvania, Philadelphia, PA 19104, United States of America; 4 Center for Neurotrauma, Neurodegeneration, and Restoration, Corporal Michael J. Crescenz Veterans Affairs Medical Center, Philadelphia, PA 19104, United States of America; 5 Laboratory of Electronics Research, Massachusetts Institute of Technology, Cambridge, MA 02139, United States of America; 6 Department of Neurology, University of Pennsylvania, Philadelphia, PA 19104, United States of America; 7 Department of Physical Medicine and Rehabilitation, University of Pennsylvania, Philadelphia, PA 19104, United States of America; 8 Department of Neurology, Drexel University, Philadelphia, PA 19104, United States of America

**Keywords:** high-density electroencephalography, dry encephalography, gel-free encephalography, signal benchmarking

## Abstract

*Objective.* To evaluate the signal quality of dry MXene-based electrode arrays (also termed ‘MXtrodes’) for electroencephalographic (EEG) recordings where gelled Ag/AgCl electrodes are a standard. *Approach.* We placed 4 × 4 MXtrode arrays and gelled Ag/AgCl electrodes on different scalp locations. The scalp was cleaned with alcohol and rewetted with saline before application. We recorded from both electrode types simultaneously while participants performed a vigilance task. *Main results.* The root mean squared amplitude of MXtrodes was slightly higher than that of Ag/AgCl electrodes (.24–1.94 uV). Most MXtrode pairs had slightly lower broadband spectral coherence (.05 to .1 dB) and Delta- and Theta-band timeseries correlation (.05 to .1 units) compared to the Ag/AgCl pair (*p* < .001). However, the magnitude of correlation and coherence was high across both electrode types. Beta-band timeseries correlation and spectral coherence were higher between neighboring MXtrodes in the array (.81 to .84 units) than between any other pair (.70 to .75 units). This result suggests the close spacing of the nearest MXtrodes (3 mm) more densely sampled high spatial-frequency topographies. Event-related potentials were more similar between MXtrodes (*ρ* ⩾ .95) than equally spaced Ag/AgCl electrodes (*ρ* ⩽ .77, *p* < .001). Dry MXtrode impedance (*x̄* = 5.15 KΩ cm^2^) was higher and more variable than gelled Ag/AgCl electrodes (*x̄* = 1.21 KΩ cm^2^, *p* < .001). EEG was also recorded on the scalp across diverse hair types. *Significance.* Dry MXene-based electrodes record EEG at a quality comparable to conventional gelled Ag/AgCl while requiring minimal scalp preparation and no gel. MXtrodes can record independent signals at a spatial density four times higher than conventional electrodes, including through hair, thus opening novel opportunities for research and clinical applications that could benefit from dry and higher-density configurations.

## 1. Introduction

Electroencephalography (EEG) is a well-known technique for non-invasive brain monitoring with applications in research and medicine. Most EEG systems use conductive gel to reduce the impedance between the scalp and the electrodes (Kappenman and Luck 2010), limiting the practicality of EEG recording. Preparing gelled systems requires anywhere from tens of minutes to over an hour of skilled preparation (Chu 2015). Moreover, the gel dries during sessions, leading to degraded signal quality (Lopez-Gordo *et al* 2014). The gel can also bridge nearby electrodes, which limits the feasibility of high-density montages (Alschuler *et al* 2014). Finally, properly removing the gel from the cap and hair is highly inconvenient, and the gels can cause skin irritation in some participants (Li *et al* 2017, Hsieh *et al* 2022).

A high-quality system that does not require gel (i.e. a ‘dry’ system) could increase the convenience and applicability of EEG. However, dry electrodes generally exhibit higher and more variable skin-electrode impedance than gelled electrodes, lowering signal quality and increasing sensitivity to artifacts (Li *et al* 2017). Due to the limitations of current dry electrodes, there has been significant focus on novel material approaches to reduce the impedance and improve the interface between the skin and the dry electrodes (Yang *et al* 2022), including materials such as platinum (Liu *et al* 2019), graphene (Shao *et al* 2019, Ko *et al* 2021, Zhai *et al* 2022), hydrogels (Alba *et al* 2010, Li *et al* 2021, 2023), and conductive textiles (Lin *et al* 2011). However, most materials have failed to gain widespread adoption due to comfort, usability, and cost issues. Additional limitations of existing dry electrode materials include difficulty and expense of manufacturing, low biocompatibility, and fragility (Radüntz 2018, Li *et al* 2020, Yang *et al* 2022). Therefore, commercially popular dry electrodes typically consist of conductive metals or polymers coated in silver, gold, Ag/AgCl, or nickel, such as g.SAHARA by g.tec GmbH and Waveguard touch by ANT Neuro (Hinrichs *et al* 2020).

To overcome the impedance limitations of existing dry electrode materials, popular dry systems have converged on the common design of a rigid frame and large textured or spiked electrodes. This design exerts high pressure on the scalp and has a large contact area per electrode, which is effective at reducing skin-electrode impedance (Li *et al* 2017, Fiedler et al 2018). However, these designs can become uncomfortable soon after application (Fiedler *et al* 2014, Li *et al* 2020). Large dry electrodes and housings are also bulky (Kübler *et al* 2014), limiting electrode density to levels that may be insufficient for more advanced analysis techniques such as source reconstruction and independent component analysis (Puce and Hämäläinen 2017, Michel and Brunet 2019). Additionally, these housings do not adequately fit all scalp sizes (Radüntz 2018) and complicate the use of EEG in the context of multimodal recording and neurostimulation techniques (e.g. EEG and transcranial magnetic stimulation).

Recently, we have demonstrated a novel material and manufacturing approach for dry EEG electrode arrays, consisting of 3D mini-pillars fabricated from Ti_3_C_2_T*_x_*-cellulose aerogels (i.e. MXtrodes). In the same work, we demonstrated the ability to record resting-state EEG with temporal and spectral characteristics comparable to gelled Ag/AgCl electrodes without applying excessive pressure, without a rigid frame, and with minimal scalp preparation. MXtrodes are safe and easy to manufacture, have excellent biocompatibility, are soft and durable, and can be scalably manufactured in high-density arrays (Driscoll *et al* 2021). These advantages address many of the limitations of existing dry technologies. However, additional rigorous quantitative evaluation of the EEG signals is needed to evaluate and benchmark MXtrodes against existing, trusted sensors. This validation will help establish MXtrodes for research and future clinical applications. Additionally, this evaluation may help support the advantages of high-density EEG configurations against conventional cm-scale single electrodes.

EEG sensors are usually validated by comparing them to a trusted standard because there is no ‘ground truth’ scalp EEG signal to serve as a baseline for evaluation (Zrenner *et al* 2020, Luck 2022). Gelled Ag/AgCl electrodes are often used as this standard (Guger *et al* 2012, Oliveira *et al* 2016, Kam *et al* 2019, Hinrichs *et al* 2020). When making these comparisons, researchers must choose whether to record each electrode type from the same location at different times or simultaneously but from different locations. We deemed simultaneous recording superior for this study because instantaneous comparisons between simultaneously recorded signals can be made. In contrast, it is only possible to compare signals recorded at different times by their average activity (Pourahmad and Mahnam 2016).

Furthermore, EEG processes are nonstationary, such that even when the underlying EEG timeseries are qualitatively different, their long-term averages can be the same (He 2014). Moreover, one important limitation of previous EEG electrode validation studies and many current dry and wet systems is that they require active pre-amplification to get high-quality timeseries (Guger *et al* 2012, Lopez-Gordo *et al* 2014, ActiCAP Slim/ActiCAP Snap—Brain Vision 2020). Many current dry EEG solutions also rely on real-time cleaning and artifact rejection to obtain usable data (DSI-24 n.d., Thirty Two Channel Wireless EEG Head Cap System—FLEX Saline n.d.). Our solution uses neither and is a true comparison to the gold standard, gelled, Ag/AgCl cup electrodes.

Therefore, to compare average and instantaneous signal similarity between and within electrode types, we recorded EEG signals from dry MXtrodes and gelled Ag/AgCl electrodes simultaneously. We investigated root mean square (RMS) amplitude, spectral power, and spectral coherence over short time windows and frequency content between electrodes. We also calculated timeseries correlations for wideband and canonical EEG narrowbands to assess the instantaneous similarity between electrodes. To explore endogenous neural signals, we computed event-related potentials (ERPs) in response to a simple vigilance task. A particular advantage of dry MXtrodes is the ease of fabricating them in high-density configurations (Driscoll *et al* 2021). Accordingly, we recorded from arrays with inter-MXtrode distances of 6 mm, less than half the spacing between electrodes in the 10-5 system (Oostenveld and Praamstra 2001), which allowed us to compare high-density EEG signals collected on MXtrodes at various inter-electrode distances.

## 2. Methods

### 2.1. Participants

The Drexel University Institutional Review Board approved the study under protocol #1904007140. We collected data from ten participants (six male). The average age of participants was 21.89 years (SD = 2.67). We recruited participants using fliers and re-contacting participants who had been enrolled in previous experiments. Participants were compensated $25 for their time. Sessions lasted approximately two hours. We excluded two participants from the analyses: one due to overall poor data quality on all channels due to a damaged adapter and another due to poor Ag/AgCl electrode signal quality, leaving eight participants in the analyses. An additional eight participants were included in the impedance sessions (described in section 2.4), and four more participants were recruited for the scalp treatment and through-hair recording study (described in section 2.11). Studies that compare basic signal properties between electrode types generally find this number of participants sufficient (Li *et al* 2020).

### 2.2. MXtrode array fabrication

**Figure 1.**
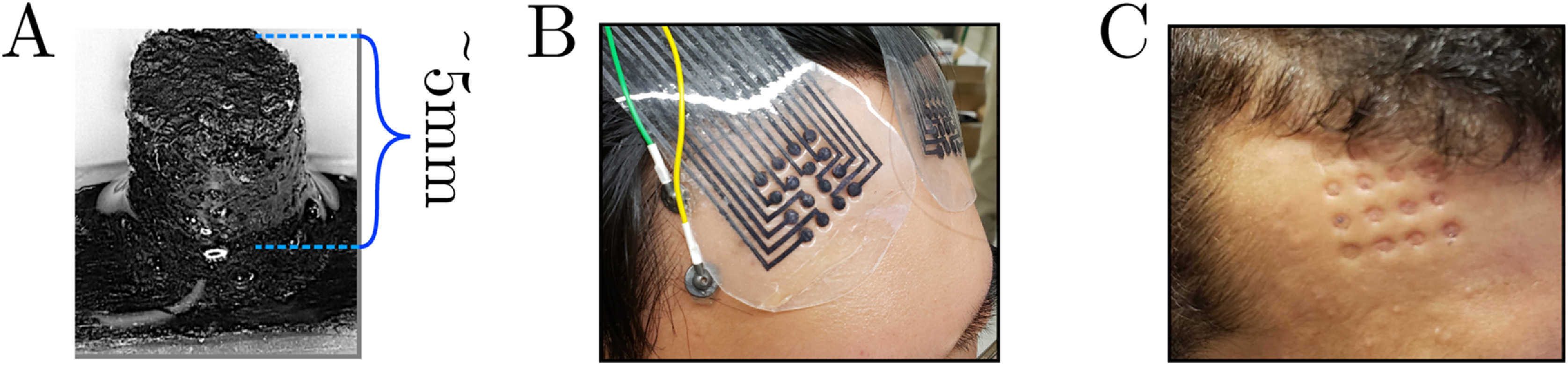
MXtrode array and scalp configuration. (A) Representative single MXtrode mini-pillar and lead embedded in PDMS. (B) Example of a high-density grid placement on F4 prior to wrapping with the elastic net. (C) Transient skin indentations after removal of the MXtrode array.

We fabricated dry EEG arrays following previously published protocols (Driscoll *et al* 2021). Briefly, we patterned the MXtrode array layout onto a nonwoven, hydroentangled cellulose-polyester blend substrate using a CO_2_ laser. We then infused the cellulose-polyester substrate by hand with a Ti_3_C_2_T*_x_* MXene dispersion at 20 mg ml^−1^ obtained from Murata Manufacturing Co. (Kyoto, Japan), which wicked into the fibers and formed a conductive composite. We fabricated the 3D mini-pillars by cutting cellulose aerogels to form cylindrical pillars, similarly infusing Ti_3_C_2_T*_x_*, and placing them at electrode locations on the patterned substrate. (figure 1(A)). After drying in a vacuum oven (80 °C, 25 mmHg), the pillars were strongly bonded to the laser-patterned substrate through MXene only (without additional adhesives). Next, we encapsulated the arrays in a ∼1 mm-thick layer of polydimethylsiloxane (PDMS), followed by degassing and curing. Finally, we trimmed the mini-pillars to a uniform height of 5 mm using a vibratome (Leica Biosystems) to expose the electrode contacts.

### 2.3. Preparation

We prepared participants’ foreheads using the method described in (Murphy *et al* 2020a): wiping the area with alcohol, gently rubbing with an exfoliating pad, and rewetting the area with 0.9% concentration saline (Cytiva). We then centered two 4 × 4 square arrays of MXtrode mini-pillar electrodes embedded in a PDMS matrix bilaterally over approximate F3 and F4 positions of the international 10-5 system (figure 1(B)). To find F3/F4, we measured the vertex position halfway from nasion to inion and marked positions 12 cm forward and 3 cm to either side from the vertex. If these positions caused the arrays to overlap the hairline, we positioned the arrays immediately below the hairline instead. We coated the PDMS border of each array in silicone spray adhesive (Hollister Adapt Medical Adhesive Spray) to keep the arrays in place temporarily. Next, we placed elastic netting (Surgilast Tubular Elastic Dressing Retainer, Size 6) over the participant’s head to hold the arrays and provide light pressure. Then, we inserted passive Ag/AgCl cup electrodes (Technomed Disposable EEG Cup Electrodes) at several positions under the netting. We placed one Ag/AgCl electrode vertically centered to the outside of each array in three of the eight participants in the analyses. In the remaining five of the eight analyzed participants, we placed two Ag/AgCl electrodes to the outside of the array instead, equally vertically spaced along it. In these participants, we placed another Ag/AgCl electrode at approximately the Iz position, which was not included in the present analyses. The setup procedure took about 5 min per participant. After removal, the MXtrodes left temporary indents (figure 1(C)). We reused four total bifrontal arrays across the eight participants. The average reuse count of the bifrontal arrays was 2.5 uses (SD = 1.64). Immediately after removal, we disinfected the arrays with alcohol wipes.

The MXtrode arrays, Ag/AgCl electrodes, and ground/reference electrodes were connected simultaneously to an Intan RHD Recording System (Intan Technologies, USA) using two RHD 32-Ch recording headstages modified to remove a short between the reference and ground, which allows for separate reference and ground electrodes. Two Natus Disposable Adhesive Disc electrodes served as ground and reference on the left and right mastoids, respectively.

### 2.4. Recording

We recorded EEG in a shielded room using a passive Intan RHD amplifier set to a sampling rate of 2 kHz. We collected the data used in our analyses as part of a larger cognitive experiment with several parts. Participants first performed 2 min blocks of alternating eyes-open and eyes-closed resting state, in which they were asked to sit quietly and remain relaxed without becoming drowsy. Participants then completed blocks of approximately 10 min of the psychomotor vigilance task (PVT) (Mentzelopoulos *et al* 2023), 12 min of the attention network task (ANT; Fan *et al* 2002), 10 min of the N-back task (Gevins and Cutillo 1993), and finally 10 additional minutes of PVT. In each trial of the PVT, a fixation cross appeared at the center of the screen. After a variable delay (2–11 s), a red dot (the probe) appeared at the center of the screen and remained for two seconds or until a button was pressed (supplementary figure 1). The next trial then began. Each 10 min block of the PVT averaged 85 trials for 170 total trials per participant. We only analyzed the first block of PVT data in this study. We chose this block to mitigate the influence of fatigue on our results (Rich *et al* 2023). The PVT task measured the simplest event-related cognitive process in the task battery, cued attention during sustained vigilance (Kribbs and Dinges 1994), and thus required the least eye movement. A single block of PVT data (10 min) is well beyond the durations commonly used for continuous comparisons (often two to four minutes) and contains enough artifact-free trials to enable event-related analyses.

We measured the MXtrode-skin impedance with the Intan RHD amplifier during the sessions. However, we found significant discrepancies between the impedance measurements from the Intan compared to a benchtop potentiostat (Gamry Ref. 600), particularly at test frequencies <100 Hz (supplementary table 1). The Intan’s minimum recommended test frequency for accurate impedance measurements is 1 kHz, which may explain the inaccuracy at lower frequencies (Foy and Harrison 2021). While 1 kHz is the appropriate test frequency for intracranial microelectrodes with impedance >103 Ω, in commercial EEG amplifiers, the test frequency is typically <100 Hz, especially for resistances approximating the target impedance for EEG electrodes (∼10 kΩ or less; Food & Drug Administration 2020, Blanch 2022, Kinnunen and Simonaho 2022). Therefore, we conducted separate impedance measurements in eight healthy volunteers (four male). The average age of participants was 26.38 years (SD = 5.36). Participants were prepared with a single MXtrode array at F3 and one or two gelled Ag/AgCl electrodes next to the array. Skin preparation was otherwise identical to that used in the EEG recording sessions. In these sessions, we measured impedance with the Gamry potentiostat at a test frequency of 10 Hz.

### 2.5. Preprocessing

We preprocessed and analyzed all data in MATLAB 2019b (Mathworks, Inc., Natick, Massachusetts, USA) using EEGLAB (Delorme and Makeig 2004), ERPLAB (Lopez-Calderon and Luck 2014), and custom functions. We imported data into MATLAB using Intan data conversion tools (‘*MATLAB RHD file reader*’) and then converted the data into EEGLAB format using custom scripts. This import included an auxiliary EEG channel, which carried analog transistor–transistor logic signals generated by the stimulus presentation software, PsychoPy^®^, to mark events (Peirce *et al* 2019). We used custom MATLAB scripts to decode event types depending on pulse width and repetition.

Following conversion to EEGLAB structures, we individually ran data from each participant through a semi-automatic preprocessing pipeline. We bandpass filtered all EEG data (Ag/AgCl and MXtrode channels) from 1 to 35 Hz using a non-causal FIR filter with a transition bandwidth of 1 Hz and cutoff frequencies (−6 dB) of 0.5 and 35.5 Hz, respectively (function ‘*pop_eegfiltnew*’; (Widmann *et al* 2015)). We selected the 1–35 Hz band to include frequencies from Delta through high Beta, which typically have a high enough signal-to-noise ratio (SNR) to be interpretable in standard-quality recordings.

We then automatically iteratively rejected MXtrode channels (function ‘*pop_clean_rawdata*’ version 2.7, using default parameters, channel rejection tool component only) with visual inspection after each round until no additional channels were rejected (supplementary figure 2). We did not include Ag/AgCl electrode data in this iterative rejection step because our analysis objective included testing the correlation between MXtrodes and Ag/AgCl electrodes, and the ‘*pop_clean_rawdata*’ function uses the correlation between nearby electrodes as a basis for rejections. Instead, we visually assessed Ag/AgCl electrodes for data quality. Next, we iteratively used the timeseries artifact rejection component of ‘*pop_clean_rawdata*’ on all channels (Ag/AgCl and MXtrodes) with default parameters, followed by visual inspection. Because we wanted to compare signals between electrode types with minimal preprocessing, we did not use the artifact subspace reconstruction feature of ‘*pop_clean_rawdata.’*

We computed the channel and timeseries rejections described above based on broadband-filtered data (1–35 Hz). We then applied these channel and timeseries rejections to copies of the data narrowband filtered to Delta (1–4 Hz), Theta (5–7 Hz), Alpha (8–12 Hz), Beta (13–30 Hz), and unfiltered data (used in spectral analyses). This process created datasets representing different frequency bands for analysis but maintained identical channels and timeseries synchronization across all versions of filtering. Filter specifications are reported in supplementary table 2.

### 2.6. Electrode selection for similarity metrics

**Figure 2.**
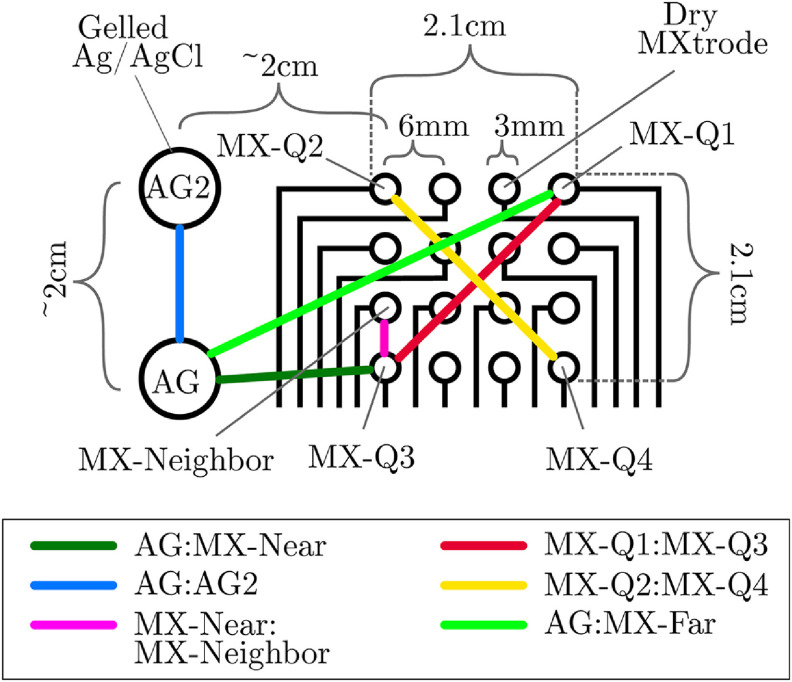
MXtrode array schematic and example comparison pairs. Layout of gelled Ag/AgCl electrodes and MXtrodes on one side of the forehead (here, the right side, centered on F4). Black lines leading from MXtrode circles are MXene-based leads embedded in PDMS. Colored lines represent pairs of electrodes used to compute similarity metrics. Identifiers for electrodes involved in comparison pairs are italicized. Colons in the legend represent electrode pair comparisons. In this case, MX-Near is the same as MX-Q3 because it was the closest MXtrode to AG. MX-Far is the same as MX-Q1 because it was the farthest MXtrode to AG. AG = primary Ag/AgCl electrode, AG2 = second Ag/AgCl electrode, MX-Near = MXtrode nearest AG, MX-Neighbor = MXtrode closest to MX-Near, MX-Far = MXtrode farthest from AG, MX-Q1 = top right corner MXtrode, MX-Q2 = top left corner MXtrode, MX-Q3 = bottom left, MX-Q4 = bottom right corner electrode.

We placed the MXtrode arrays and Ag/AgCl electrodes at different scalp locations in our design (figure 1), which may have led to recording slightly different sources of brain activity (Michel and Murray 2012). One method for handling this potential confound is to compute a comparison between two trusted sensors to serve as a baseline for the expected differences in each metric (Lopez-Gordo *et al* 2014). We used a pair of Ag/AgCl electrodes for this baseline. We also compared signals between a MXtrode:Ag/AgCl pair to test inter-electrode type differences and several pairs of MXtrodes to test inter-MXtrode differences at various distances (figure 2). We selected six pairs of electrodes for analysis. We chose five of these pairs to replicate the densest spacing in a standard 10-5 array (approximately 2 cm; Oostenveld and Praamstra 2001) within and between electrode type. We chose the sixth pair, which compared two MXtrodes at 6 mm spacing, to explore how signal similarities and differences change at the extreme densities possible with the MXtrode array. No Ag/AgCl comparison pair could be formed at this density because Ag/AgCl electrodes cannot be placed much closer than 2 cm before bridging becomes difficult to avoid. To select electrodes for each of these comparisons, first, we selected the lowest impedance Ag/AgCl electrode, the ‘primary’ electrode, and labeled it ‘AG’. We compared AG to (a) the second Ag/AgCl electrode (‘AG2’) in cases where it was available (*n* = 9), forming the ‘AG:AG2’ pair, (b) the MXtrode nearest to AG, which varied across participants (‘MX-Near’) forming the ‘AG:MX-Near’ pair, and (c) the MXtrode farthest from AG, which varied across participants (‘MX-Far’) comprising ‘AG:MX-Far’ pair. We compared the MX-Near electrode to the MX nearest to it (‘MX-Neighbor’), forming the ‘MX-Near:MX-Neighbor’ pair. Finally, we compared the MXtrodes at the corners of the array (the furthest possible distances within the array) to the top right ‘MX-Q1’ and bottom left ‘MX-Q3’ corner MXtrodes (forming the ‘MX-Q1:MX-Q3’ pair) and finally the top left ‘MX-Q2’ and bottom right ‘MX-Q4’ corner MXtrodes (forming the ‘MX-Q2:MX-Q4’ pair). Across arrays, the MXtrodes involved in each pair sometimes differed slightly due to channel rejection (supplementary figure 2). If we rejected the MXtrode that would normally have been used in any comparison for poor signal quality, we used the next closest MXtrode. We did not reject any Ag/AgCl electrodes for the retained participants.

### 2.7. Timeseries analyses

Using the electrode pairs described above, we first computed timeseries correlations for all narrowband and broadband data (MATLAB function ‘corr’). Correlation is insensitive to discontinuities because it ignores temporal ordering. Therefore, we computed correlations after artifact rejection but without epoching or rejection sections of data with discontinuities.

### 2.8. Spectral and RMS analyses

We next extracted epochs for spectral and RMS analysis. Fourier spectral analysis requires epochs of sufficient length to characterize the lowest frequency included in the transform; at least two cycles are required, and four are recommended (Luck 2014). Therefore, we chose to limit our spectral analyses to a lower-bound frequency of 2 Hz, which implies extraction of 2-second epochs to meet the recommended number of cycles. This epoch length achieves a good trade-off between the lowest analyzable frequency and the amount of data to be included in the analysis. Based on this rationale, we extracted all possible continuous non-overlapping 2-second epochs from each recording. Like the channel and timeseries rejections, epoch extraction was identical for all versions of filtering. These 2-second epoched datasets (‘regularly epoched data’; *M* = 100.87 epochs, SD = 46.63) were used as a basis for all further spectral and RMS analyses, except for permutation analysis.

We computed spectral power on the unfiltered version of the regularly epoched data by running the EEGLAB function ‘spectopo’ on each two-second epoch with the parameters 50% hamming window overlap, a range of 2–35 Hz with 1 Hz frequency resolution. We additionally computed spectral coherence (MATLAB function ‘mscohere’ with the same parameters used for spectopo) between each electrode pair and RMS amplitude for each electrode included in the analysis. For all three metrics, we computed values within each epoch, averaged across epochs within participant, and then grand averaged across participants.

### 2.9. ERP analyses

We additionally computed ERPs time-locked to PVT probe events in broadband filtered data (1–35 Hz). The epochs were −200–1000 ms event-locked to PVT cues (‘cued epochs data’; *M* = 19.6 epochs per participant, SD = 8.89). We baselined the epochs from −200 to 0 ms. We extracted epochs for electrodes involved in the AG:AG2, AG:MX-Near, and MX-Near:MX-Neighbor in ERP analyses. We also added the MX-Corner1:MX-Corner2 comparison (comprising the MX-Q1:MX-Q3 and MX-Q2:MX-Q4 pairs when available). We combined these comparisons because both corner pairs had the same distance between the electrodes. We computed values within each epoch, averaged across epochs within participant, and then grand averaged across participants.

### 2.10. Permutation analyses

For timeseries correlation and spectral coherence metrics, we performed a permutation analysis on a broadband-filtered version of the cued epoch data. To ensure a baseline level of exchangeability in the data epochs, we used event-locked PVT-cue epochs. We permuted the epoch order of the AG and MX-Near electrode 1000 times within-participant. We then recalculated within-participant average timeseries correlation and spectral coherence within all three possible pairs of original and permuted data, AG:MX-Near (Permuted), AG:AG (Permuted), and MX-Near:MX-Near (Permuted).

### 2.11. Effect of skin preparation and through-hair recordings

We performed one additional experiment to evaluate the performance of the MXtrode arrays of the same fabrication described above in conditions where (1) there was no scalp preparation and (2) through diverse hair types. We collected data from four additional participants (2 female). The average age was 27.25 years (SD = 6.55). Participant demographics and hair characteristics are listed in table 1.

**Table 1.** Individual demographics and hair characteristics.

Participant	Ethnicity	Sex	Hair color	Hair shape	Hair strand thickness	Hair follicle density	Hair length
1	Western Caucasian	Male	Silver	Straight	Fine	Sparse	<1 cm
2	Eastern European Caucasian	Male	Brown	Wavy	Coarse	Dense	<1in
3	Mediterranean Caucasian	Female	Brown	Straight	Medium	Dense	Shoulder length
4	African American	Female	Brown	Curly	Coarse	Dense	Past the shoulder

Sessions lasted approximately two hours. We recorded EEG using the same Intan RHD system in the same shielded room with the same settings. Participants performed two rounds of the same PVT task described above with impedance recorded before, between, and after the PVT runs (separated by approximately 45 min apiece). Impedance on 4 MXtrodes per array and all 6 Ag/AgCl electrodes was recorded on each electrode individually at three time points using the Gamry potentiostat. For each participant, we recorded three new conditions simultaneously: (a) frontal F4 location with full prep (impedance only due to hardware limitations per below and to reduce burdens to participants), (b) frontal F3 location with no prep, (c) Cz-centered location with prep, but through hair. See supplementary figure 3.

For (a), we used the exact procedure explained in section 2.3. However, we only applied this procedure to a single 4 × 4 MXtrode array on the F4 location instead of two arrays placed on F3 and F4. In all cases, two Ag/AgCl electrodes were placed lateral to the array. For (b), the location and procedure were identical to the procedure explained in section 2.3 above. However, we did not wipe the area with an alcohol wipe, exfoliate it, nor re-wet it with saline. In all cases, two Ag/AgCl electrodes were placed lateral to the array. For (c), we found the Cz location along the vertex of the participant’s hair. We then parted the participant’s hair along that location. Next, we wiped the area with an alcohol wipe, allowed it to dry, and rewet with saline. We then placed a 4 × 4 MXtrode array so that the four MXtrodes in the leftmost column were directly on the visible line of the scalp where we parted the hair. We placed two gelled Ag/AgCl electrodes immediately to the left and filled them with gel. We then stretched medical wrap over the array, around the head, under the chin, and around once more to ensure enough pressure for the MXtrodes to contact the scalp. See supplementary figure 3 for an illustration of the session set-up for the cranial vertex site. The (b) F3 no-preparation and (c) hairy Cz-centered arrays were connected to the Intan with the same ground and referencing as described above. The F4 array for condition (a) was not connected to the Intan due to limitations in our custom adapters. We only used it to record impedance values.

EEG was preprocessed as described in 2.5. Electrode selection was as described in 2.6. For the vertex, however, we only collected data on 4 MXtrodes out of the 16-Mxtrode array in which we focused on achieving adequate scalp contact through the hair. This limited pairings to AG:MX-Near, AG:AG2, MX-Near:MX-Neighbor, and AG:MX-Far. The distance between AG:MX-Far was also slightly reduced due to the geometry. Timeseries, spectral, and RMS analyses were conducted as described in 2.7 and 2.8, respectively.

## 3. Results

### 3.1. Impedance distribution on the MXtrode and Ag/AgCl electrodes

**Figure 3.**
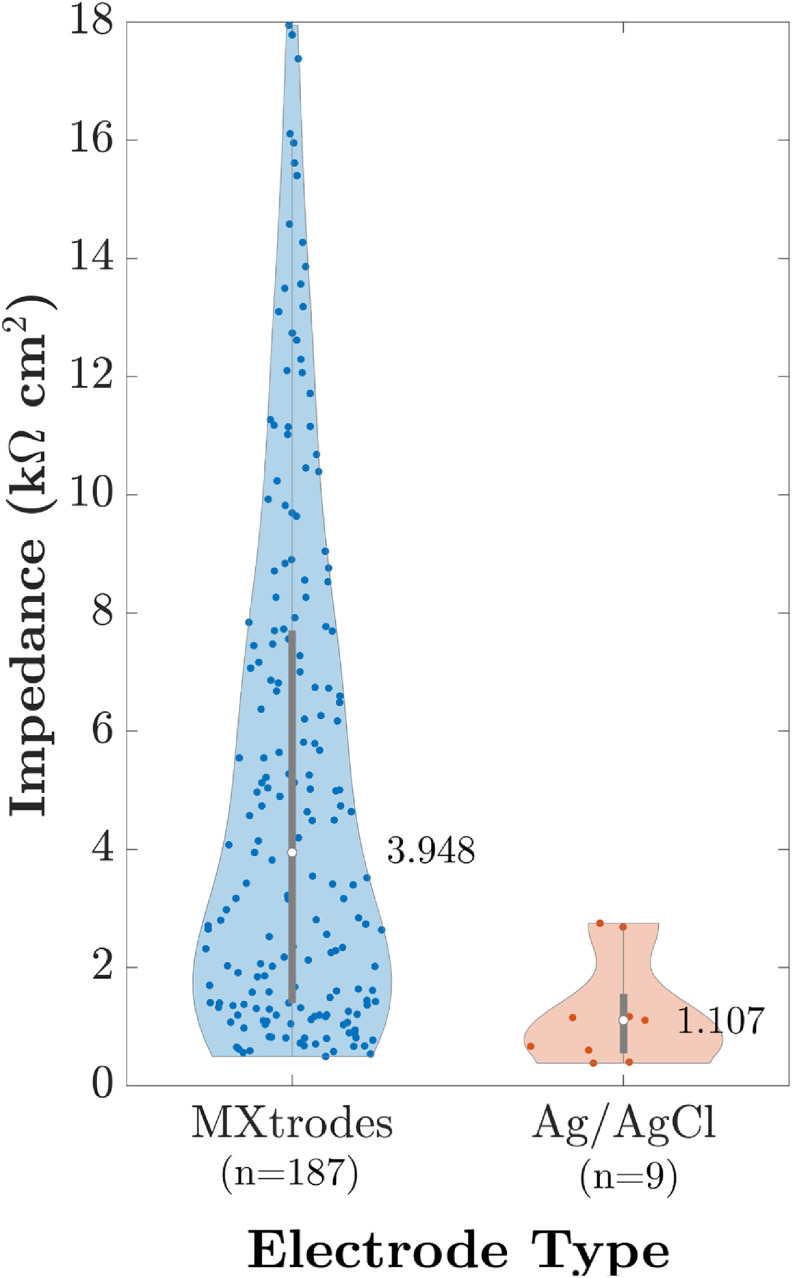
Area-normalized impedance. MXtrodes values are pooled from all electrodes available in the impedance sample. Ag/AgCl values are the impedance of the primary Ag/AgCl electrode (‘AG’) for each participant from the impedance sample. The median impedance of each electrode type is listed next to each violin-plot and is represented with a white dot. Individual measurements are represented as colored dots inside the violin-plot, with the central grey rectangle and line depicting a standard box plot. *n* = number of electrodes.

We compared the area-normalized impedances of Ag/AgCl electrodes and MXtrodes (figure 3). Standard outlier detection led to 1/10 Ag/AgCl electrodes and 13/200 MXtrodes rejected from the data. The impedance on the Ag/AgCl electrodes was lower (median = 1.107 kΩ cm^2^, interquartile range = 1.000 KΩ cm^2^, *n* = 9 electrodes) than MXtrode impedance (median = 3.948 kΩ cm^2^, interquartile range = 6.298 KΩ cm^2^, *n* = 187 MXtrodes). The difference in impedance between the electrodes was significant (Mann–Whitney *U* = 262, *n*_1_ = 187, *n*_2_ = 9, *p* < .001). See supplementary figure 4 and supplementary table 3 for additional impedance data suggesting good MXtrode stability over time, including moderate drops in impedances over time, likely due to the influence of sweat (Murphy *et al* 2020b).

### 3.2. Timeseries-based metrics

**Figure 4.**
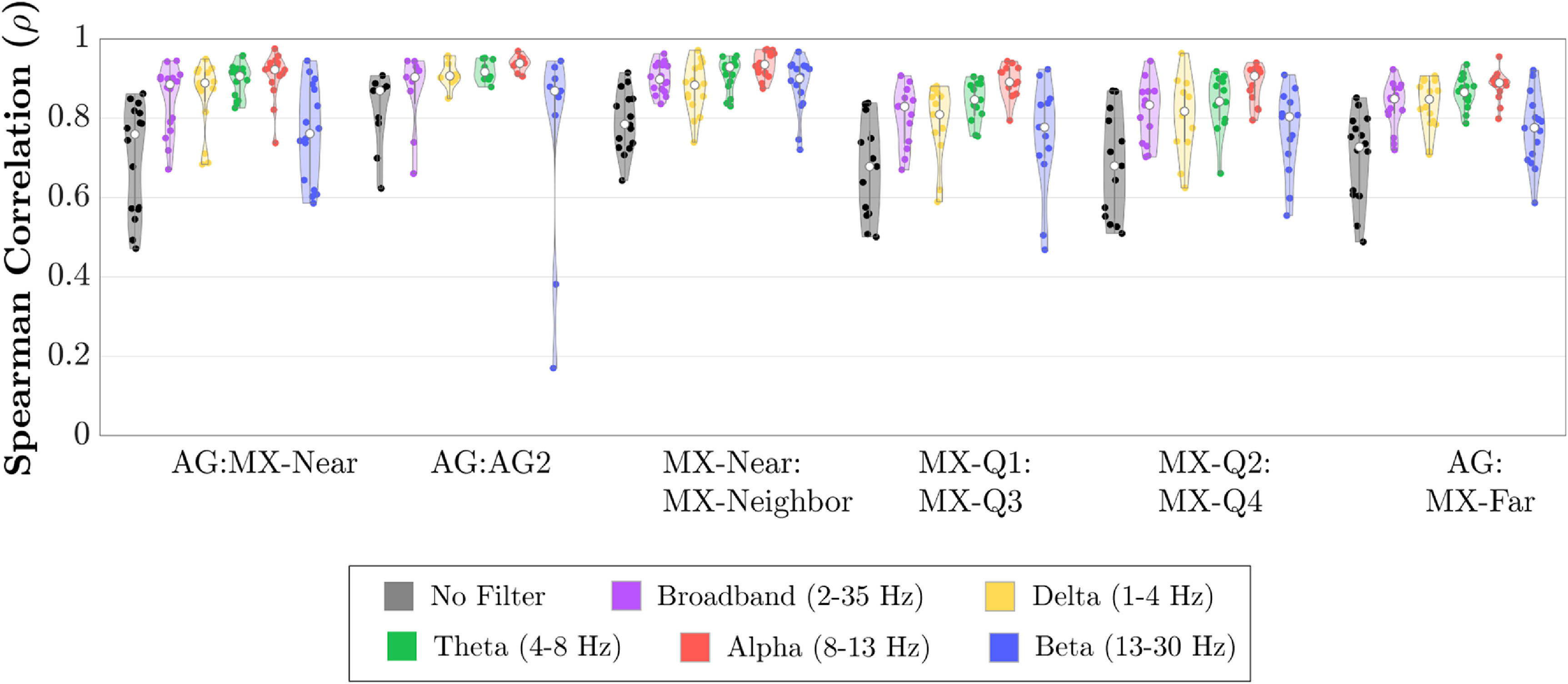
Spearman’s rank correlations between time courses from electrode pairs. The mean correlation across arrays was computed for each electrode pair in the unfiltered time series. Broadband (1–35 Hz), Delta (1–4 Hz), Theta (4–8 Hz), Alpha (8–13 Hz), and Beta (13–30 Hz) bands. The median correlation of each electrode comparison is represented with a white dot. Individual correlations are represented as colored dots inside the violin-plot. Numerical values are reported in supplementary table 4. All correlations were significant (*p* < .001). AG = primary Ag/AgCl electrode, AG2 = second Ag/AgCl electrode, MX-Near = MXtrode nearest AG, MX-Neighbor = MXtrode closest to MX-Near, MX-Far = MXtrode farthest from AG, MX-Q1 = top right corner MXtrode, MX-Q2 = top left corner MXtrode, MX-Q3 = bottom left, MX-Q4 = bottom right corner electrode.

Metrics computed from timeseries data included RMS amplitude (table 2) and Spearman’s rank correlation (figure 4 and supplementary table 4). The broadband RMS amplitude was higher on all MXtrodes than on Ag/AgCl electrodes. Spearman’s rank correlations revealed high overall similarity within and between electrode types. Correlations were highest in the Alpha band and for the MX-Near:MX-Neighbor and AG:AG2 pairs. The Delta and Beta bands observed the lowest correlations, especially for the MX-Near:MX-Far and MX-Near:AG pairs. Correlations for all participants in all electrode pairings by frequency bands were significant (*p* < .001). See supplementary table 5 for results reporting high split-half correlations between timeseries, supplementary figures 5 and 6 for timeseries correlations from no-preparation and hairy site electrodes, and supplementary tables 6 and 7 for RMS results from no-preparation and hairy site electrodes.

**Table 2.** Broadband RMS amplitude for MXtrodes recorded from F3 & F4 and Ag/AgCl electrodes lateral to the arrays. AG = primary Ag/AgCl electrode, AG2 = second Ag/AgCl electrode, MX-Near = MXtrode nearest AG, MX-Neighbor = MXtrode closest to MX-Near, MX-Q1 = top right corner MXtrode, MX-Q2 = top left corner MXtrode, MX-Q3 = bottom left, MX-Q4 = bottom right corner electrode.

Electrode	AG	MX-Near	AG2	MX-Neighbor	MX-Q1	MX-Q2	MX-Q3	MX-Q4
*n*	16	9	13	13	16	16	16	16
*M*	9.46	10.41	8.86	10.30	9.70	9.71	9.71	10.80
SD	2.49	3.38	1.69	3.27	1.50	2.23	2.05	3.44

*n* = number of electrodes, *M* = mean, SD = standard deviation.

### 3.3. Spectral metrics

**Figure 5.**
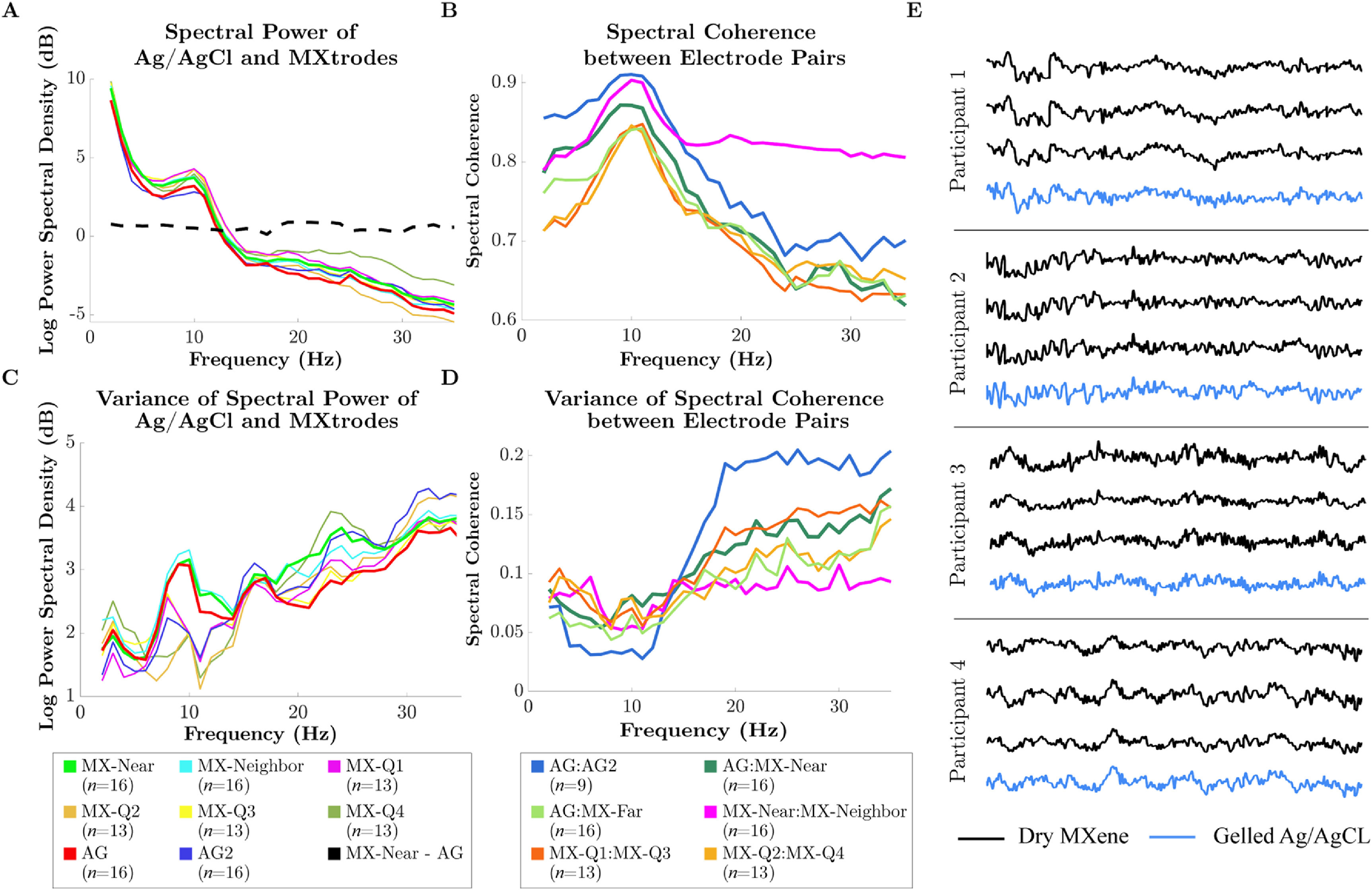
Spectral characteristics within and between MXtrodes and Ag/AgCl electrodes. (A) Log spectral power of MXtrodes and Ag/AgCl electrodes across the 1–35 Hz frequency range averaged by frequency across arrays of a given electrode type. *n* = number of electrodes. (B) Spectral coherence between sensors across arrays of a given electrode type per frequency. Note that the vertical axis begins at 0.6. *n*= number of electrode pairs. (C) Variances of the log spectral power across arrays of a given electrode type per frequency. *n* = number of electrodes. (D) Variances of the spectral coherence across arrays of a given electrode type per frequency. *n* = number of electrode pairs. AG = primary Ag/AgCl electrode, AG2 = second Ag/AgCl electrode, MX-Near = MXtrode nearest AG, MX-Neighbor = MXtrode closest to MX-Near, MX-Far = MXtrode farthest from AG, MX-Q1 = top right corner MXtrode, MX-Q2 = top left corner MXtrode, MX-Q3 = bottom left, MX-Q4 = bottom right corner electrode. (E) Representative EEG time series segment for each participant across three MXene electrodes and one gelled Ag/AgCl electrode. All electrodes were from the right (F4) side of the scalp.

Metrics derived from spectral transformations included spectral power and spectral coherence (figure 5). The MX electrodes had higher spectral power by 1 dB or less than the AG electrode at all frequencies (figure 5(A)). Spectral power was similar across electrodes otherwise, with the greatest absolute differences between about 1–10 Hz and 17–25 Hz. The expected Alpha-band power enhancement was present on all the electrode types.

Spectral coherence was high overall and highest in the Alpha band for all pairs (figure 5(B)). Closely spaced MXtrodes (MX-Near:MX-Neighbor) were slightly less coherent in lower frequencies but much more coherent in higher frequencies than the further-spaced Ag/AgCl electrode pair (AG:AG2). MXtrode pairs spaced similarly to Ag/AgCl electrode pairs (MX-Near:MX-Far pairs) had slightly lower coherence across all frequencies, especially in lower frequency bands. The split-half signal stability of spectral coherence was high and is reported in supplementary table 8. Qualitatively, the EEG timeseries of the Ag/AgCl electrodes and MXtrodes within each individual were highly similar (figure 5(E)).

**Figure 6.**
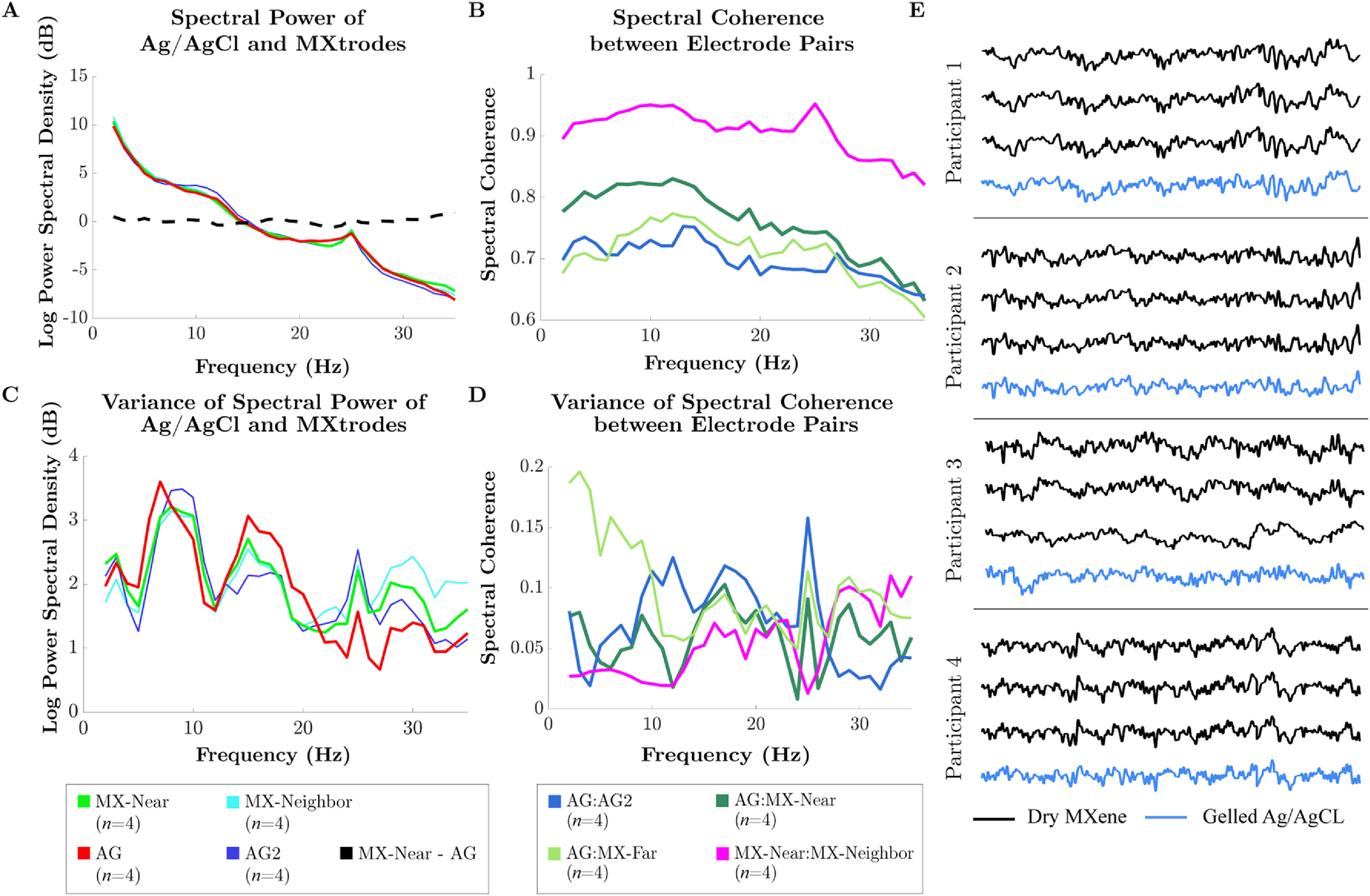
Spectral characteristics within and between MXtrodes at the cranial vertex (hairy site) and Ag/AgCl electrodes. (A) Log spectral power of MXtrodes and Ag/AgCl electrodes across the 1–35 Hz frequency range averaged by frequency across arrays of a given type. *n* = number of electrodes. (B) Spectral coherence between sensors across arrays of a given type per frequency. Note that the vertical axis begins at 0.6. *n* = number of electrode pairs (C) variances of the log spectral power across arrays of a given type per frequency. *n* = number of electrodes (D) variances of the spectral coherence across arrays of a given type per frequency. *n* = number of electrode pairs AG = primary Ag/AgCl electrode, AG2 = second Ag/AgCl electrode, MX-Near = MXtrode nearest AG, MX-Neighbor = MXtrode closest to MX-Near, MX-Far = MXtrode farthest from AG. (E) Representative EEG time series segment for each participant across three MXene electrodes and one gelled Ag/AgCl electrode.

Figure 6 shows the performance of the arrays through diverse hair types at site Cz. Overall, we recover similar 1/*f* signals and evidence of modest group level but detectable alpha power elevations in 3 out of 4 individual participants (supplementary figure 7). Moreover, good qualitative correspondence between the timeseries of the Ag/AgCl electrodes and MXtrodes within each individual was again observed again in these data (figure 6(E)). For spectral metrics in a no-preparation forehead site, see supplementary figure 8.

### 3.4. ERP analysis

**Figure 7.**
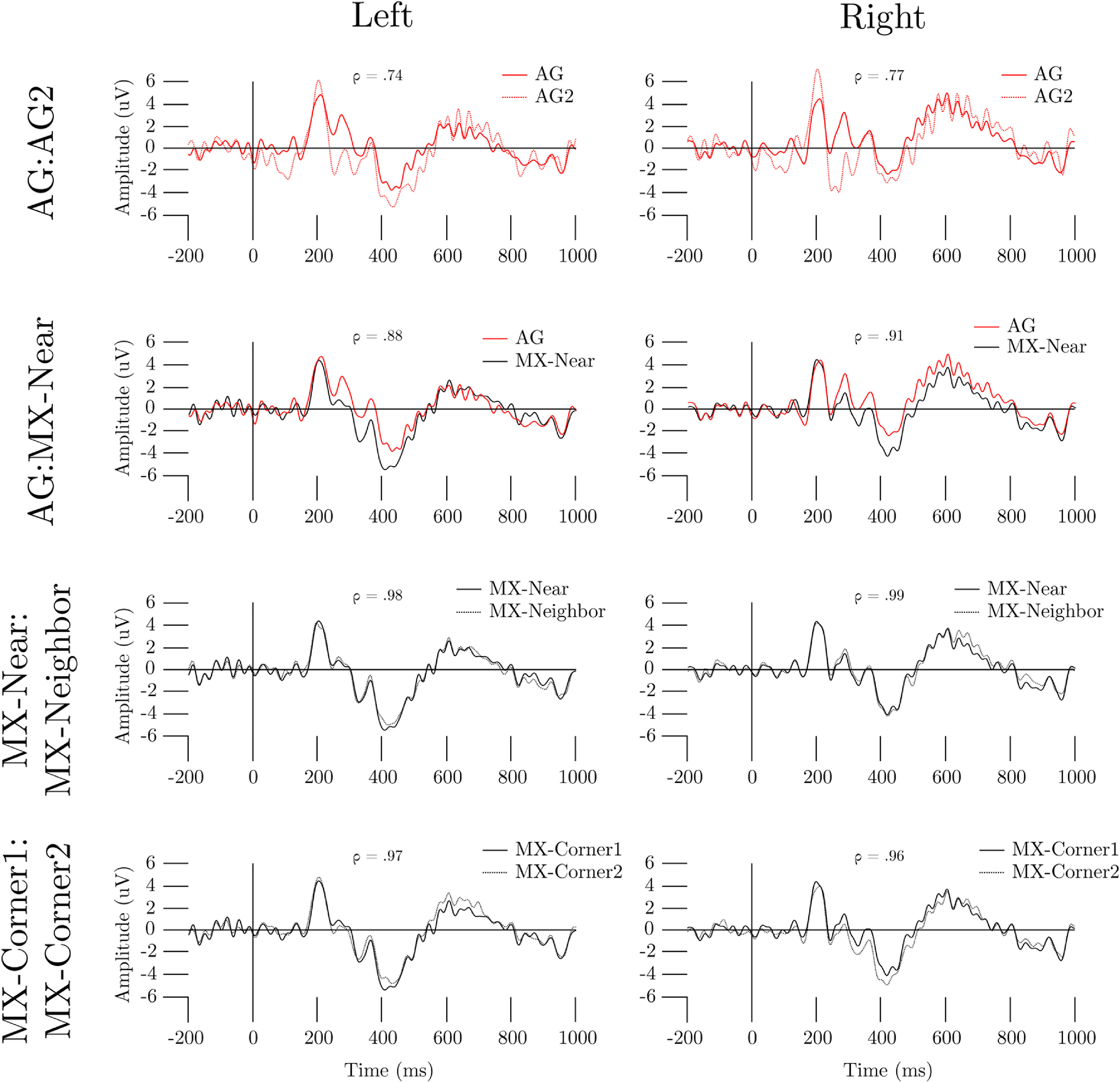
ERPs of MXtrodes and Ag/AgCl electrodes. Grand average ERPs are overlaid in the pairs AG:AG2, AG:MX-Near, MX-Near:MX-Neighbor, and MX-Corner1:MX-Corner2 (the pair combining the MX-Q1:MX-Q3 and MX-Q2:MX-Q4 pairs), for the right and left (F3 and F4 centered, respectively) sides of the head. Spearman’s ρ for each comparison is reported on each panel. AG = primary Ag/AgCl electrode, MX-Near = MXtrode nearest AG, MX-Neighbor = MXtrode closest to MX-Near, MX-Far = MXtrode farthest from AG.

ERPs of the cue event derived from 1 to 35 Hz data reveal high similarity within and between electrode types (figure 7). All electrodes exhibit clear P200 and N400 components. The largest absolute deviations in ERP amplitude in both hemispheres occurred between the AG and AG2 electrodes. Spearman’s rank correlations of all ERPs were significant (*p* < .001). The AG:AG2 pair had the weakest correlation, and the MX-Near:MX-Neighbor pair had the strongest.

### 3.5. Cued-epoch permutation analysis

**Figure 8.**
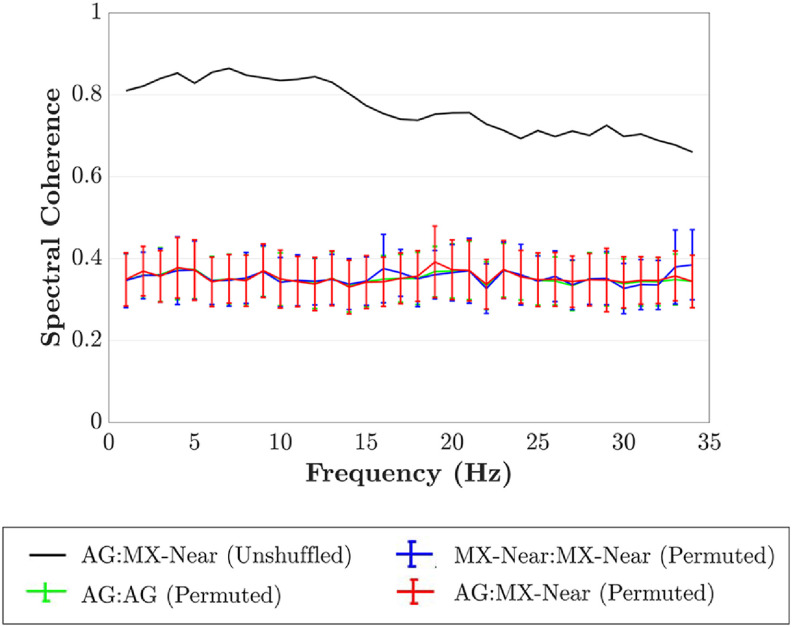
Permutation analysis of broadband event-related spectral coherence. Spectral coherence computed from epochs event-locked to PVT cues. The error bars represent the standard deviation of values across all permutations. AG = primary Ag/AgCl electrode, MX-Near = MXtrode nearest AG, MX-Neighbor = MXtrode closest to MX-Near, MX-Far = MXtrode farthest from AG.

The correlation and spectral coherence between the non-permuted AG:MX-Near cued-epoch data was very high. The permuted AG:MX-Near cued-epoch data revealed that event-related signal content induced some broadband spectral coherence between electrodes. However, non-permuted correlation and spectral coherence between electrode pairs were always much higher than the permuted comparisons (table 3; figure 8).

**Table 3.** Permutation analysis of broadband event-related Pearson’s correlation. For the permuted values, each participant contributed 10 000 permutations. AG = primary Ag/AgCl electrode, MX-Near = MXtrode nearest AG.

Electrode pair	AG:MX-Near (Unshuffled)		AG:MX-Near (Permuted)		AG:AG (Permuted)		MX-Near:MX-Near (Permuted)	
*n*	10		10		10		10	
	*M*	SD	*M*	SD	*M*	SD	*M*	SD
Correlation (2-35 Hz)	0.84	0.08	0.03	0.02	0.03	0.02	0.04	0.03

*n* = number of participants, *M* = mean, SD = standard deviation.

## 4. Discussion

In this study we quantified the similarity of scalp EEG signals recorded simultaneously from dry high-density MXtrode electrodes and gelled Ag/AgCl electrodes. Across all the metrics we computed, scalp EEG signals recorded on dry Mxtrode channels were highly similar to signals on gelled Ag/AgCl in the 1–35 Hz range. The most notable deviations in individual frequency bands were slightly lower correlation and spectral coherence between pairs involving MXtrodes in the Delta through Alpha-bands and much higher correlation and spectral coherence between the nearest MXtrode channels in the Beta-band. The signals collected on Mxtrode channels revealed Alpha-band power and ERP signatures of cortical origin. Participants tolerated the Mxtrode arrays well. No participants reported discomfort due to the array or wrapping, even when asked at the end of the session. Overall, our results support that MXtrodes record similar average and instantaneous spectral and timeseries information as Ag/AgCl electrodes and are suitable general replacements, including through the hair, when adequate scalp contact is achieved.

We performed all analyses on both Ag/AgCl and Mxtrode timeseries so that Ag/AgCl results could serve as a baseline to compare Mxtrode signals. These comparisons revealed high similarity across the electrode types. We found that RMS amplitudes were slightly higher on dry MXtrodes compared to a nearby Ag/AgCl electrode. The power spectral density analysis clarified that this amplitude difference was a relatively uniform difference of <1 dB across the 1–35 Hz range (figure 5(A)). This power difference had little impact on the parity of signals recorded across the electrode types because it was similar across all frequencies. In addition, we observed only slightly lower timeseries correlations and spectral coherence between MXtrodes and Ag/AgCl electrodes than between two Ag/AgCl electrodes. The magnitude of the timeseries correlation and spectral coherence for all electrode pairs remained at high levels for inter-electrode comparisons (figure 4) (Li *et al* 2020). Together, these findings suggest that MXtrodes and Ag/AgCl electrodes record qualitatively similar timeseries and spectral content when positioned at similar distances.

We detected signatures of cortical origin in the spectral features of the Alpha-band on both electrode types. Specifically, we observed a peak in Alpha band spectral power (figure 5(A)), a well-known signature of occipital brain sources (Smith *et al* 2017) on both electrode types. We also observed a peak in spectral coherence in the alpha band for all electrode pairs (figure 5(B)). The fact that both spectral power and coherence peaked in the Alpha-band suggests that these results might be linked. The likely source of this link is phasic alpha bursting (Rusiniak *et al* 2018). Coherence measures the stability of the relative phase and amplitude between two timeseries and does not inherently encode frequency power information. However, amplitude covariation between timeseries has been linked to increased coherence (Lachaux *et al* 1999) and is a defining feature of alpha bursts. Therefore, we expect that our results encode the presence of alpha bursts as both enhanced average spectral power over the recording and enhanced spectral coherence due to the shifts in amplitude that bursts induce. These results emphasize that MXtrodes detect cortical signals similar to gelled Ag/AgCl electrodes. The preserved spectra and ability to detect individually variable alpha peaks (Bazanova and Vernon 2014) with MXtrodes through the hair when they are observed in Ag/AgCl recordings reveals that they could be deployed in full-head montages, such as in full EEG cap designs.

Where we did observe differences in spectral coherence between electrode pairs, those differences were likely related to electrode geometry and spacing. First, we found increased low-frequency (Delta through Alpha-band) coherence on Ag/AgCl electrodes compared to MXtrodes. This difference existed across all electrode pairs that involved a Mxtrode, which strongly suggests an effect of electrode type. A likely explanation for this finding is that the larger diameter and broad gel base of the Ag/AgCl electrodes spatially integrate signals from a larger scalp area than the smaller dry contacts. A larger area spatially low-passes scalp information, which reduces the aliasing of higher frequency information into lower frequencies (Iivanainen *et al* 2020), effectively reducing noise. This noise reduction may have increased low-frequency coherence on gelled Ag/AgCl electrodes relative to the smaller MXtrodes. Lower EEG frequencies also have lower spatial frequency (Burgess and Gruzelier 1997, Srinivasan *et al* 1998), meaning less information is lost over the larger Ag/AgCl spatial integration area. These results suggest that small contact-area electrode geometries, such as those we used in our Mxtrode arrays, are slightly less optimal for measuring low-frequency spectral information. However, even the extremely small 3 mm diameter electrodes we used resulted in only moderate decreases in low-frequency coherence. Additionally, it is possible to fabricate MXtrodes at larger diameters with minimal modification to current manufacturing processes (Driscoll *et al* 2021).

Second, we observed that the nearest MXtrodes (the MX-Near:MX-Neighbor pair) had much higher spectral coherence and timeseries correlation than any other pair of electrodes in the Beta-band. Because we did not observe these increases on MXtrode pairs at larger distances, they likely reflect that the MX-Near:MX-Neighbor pair sampled high-frequency topographies more densely. High EEG frequency bands such as Beta have higher spatial-frequency scalp topographical features than lower frequencies (Burgess and Gruzelier 1997, Srinivasan *et al* 1998). Fully characterizing higher spatial-frequency information requires denser sampling (Kuhnke *et al* 2018). Thus, our results suggest that high-density arrays may be especially useful for detecting the topography of high-frequency scalp signals. Although our arrays did not provide full-head coverage, we suggest that it is possible that our results would generalize across the scalp. Different scalp locations have different SNRs within frequency bands, which can influence correlation and coherence measurements. However, our frontal arrays were maximally distant from the brain’s strongest rhythm (occipital Alpha), and the intrinsic rhythm of the frontal region is a relatively weak Theta signal. Therefore, the frontal scalp is an appropriate initial location to examine.

Importantly, not all scalp topographical information derives from brain sources. Environmental noise, equipment noise, and non-brain physiological artifacts can contaminate the scalp EEG signal. High-frequency bands (Beta and above) are particularly susceptible to contamination since their SNR over background 1/*f* activity is already low (Muthukumaraswamy 2013). Our data cannot confirm that the enhanced high-frequency coherence we observed between nearby MXtrodes originates from brain sources rather than artifacts. However, we can draw several conclusions. First, the MX-Near:MX-Neighbor pair’s high Beta timeseries correlation and spectral coherence are necessarily driven by a shared signal rather than a signal unique to individual MXtrodes, so it is unlikely to represent manufacturing variability in MXtrodes.

Furthermore, we observed that MXtrodes spaced at a similar distance as the AG:AG2 pair (∼2 cm) have very similar correlation and coherence to the AG:AG2 pair. The only standout difference is the elevated high-frequency correlation and coherence on closely spaced MXtrodes. These findings suggest that the enhanced high-frequency similarity in the MX-Near:MX-Neighbor pair is likely due to their closer spacing rather than systematic differences in signal content compared to Ag/AgCl electrodes. Thus, while we cannot rule out artifact sources in our data, our results demonstrate the potential for enhanced spatial resolution to reveal previously unmeasurable topographical features, provided that one can separate them from artifacts.

It should be noted that potential scalp location effects in our analysis might have prevented a fully balanced comparison across electrode types. However, we expect that scalp location effects were small. Scalp signals in most frequency bands are quite similar at sensors spaced 2 cm apart (Srinivasan 2005). Any true scalp location-based differences would manifest most clearly in the Beta band because higher frequency topographies have EEG sources with both more real variation and weaker signals (Zelmann *et al* 2014). Indeed, in all comparisons, we measured the lowest coherence and correlation in the Beta band. This finding also emphasizes the information gained by the ultra-high density MXtrode array, which had a much smaller Beta band drop-off in these metrics.

To further clarify whether the sensors included in our analysis recorded similar brain-sourced signals, we computed ERPs, which aggregate event-locked scalp activity and average out environmental noise (Luck 2014) thus restricting the analysis to only signals originating in the brain. The PVT cue event ERPs computed from MXtrodes and Ag/AgCl electrodes were qualitatively similar (figure 7). All ERPs exhibited clear P200 and N400 components. The P200-N400 complex is a set of frontal components commonly observed in response to visual stimuli and attentional demand (Kanske *et al* 2011). Furthermore, we observed the largest ERP amplitude differences between the AG:AG2 pair in either hemisphere. These findings suggest that the differences in ERP amplitude between other pairs of electrodes (i.e. MXtrodes) are smaller than what could be attributed to a 6 mm shift in electrode position. The potential scalp location effects could also cause the lower ERP correlation in the AG:AG2 condition compared to the similarly-spaced MX-Corner1:MX-Corner2 condition. Alternatively, this difference in correlation could reflect that the gel base of the Ag/AgCl electrodes have less standardized scalp contact areas than MXtrodes and, thus, slightly less similar broadband signal content. Our data cannot adjudicate between these possibilities. However, because it is unlikely that we recorded extremely similar ERPs in the MX-Corner1:MX-Corner2 comparison by chance, our results support at least the non-inferiority of MXtrodes relative to Ag/AgCl electrodes for recording ERPs.

In event-related recordings, signals may derive similarity from both the standardized ‘average’ ERP brain response induced by the stimulus and the instantaneous activity unique to each trial. Our permutation analysis tested how much of the similarity between MXtrodes and Ag/AgCl electrodes in event-related recordings (the PVT cue event) was due to average versus instantaneous activity. The correlation and coherence of the permuted epochs were much lower than that of the non-permuted epochs across all frequencies (table 3; figure 8). Therefore, instantaneous activity was responsible for most AG:MX-Near correlation and coherence as opposed to average event-related induced activity. This finding suggests that MXtrodes recorded similar average brain responses to Ag/AgCl electrodes and similar instantaneous activity. Researchers can therefore interpret MXtrode timeseries similarly to Ag/AgCl timeseries.

Another common artifact class of concern in high-density arrays is bridging. Bridging is when a conductive medium links two electrodes, causing their signals to become identical and non-comparable to the rest of the array. Dry electrodes experience this issue less commonly than gelled electrodes since there is no gel to smear between electrodes accidentally but sweat or remaining saline after scalp rewetting could still have bridged the MXtrodes in our arrays. However, bridged signals are nearly identical across all frequency bands (Alschuler *et al* 2014). We did not observe identical signals on any electrode pairs, so bridging was not a large concern, even with our quick scalp preparation method.

Overall, the strong similarity between dry MXtrodes and gelled Ag/AgCl electrodes, including through diverse hair types, across the metrics we computed is striking, especially considering that MXtrodes had higher average area-normalized impedance than gelled Ag/AgCl electrodes in a separate test (figure 3 and supplementary table 3). The larger variance we observed in MXtrode impedance may be partially due to fabrication variability or participant-wise skin properties, which have a greater impact on dry electrodes than on gelled electrodes (Li *et al* 2017). Additionally, the impedance of dry electrodes tends to drift more over time (Krachunov and Casson 2016). Multi-material strategies could improve the performance of MXtrodes. Pure-MXene leads have advantages in scalability, but future work should explore whether coating the electrode contact area with conductive polymers such as PEDOT can improve impedance (Donahue *et al* 2020).

The MXtrode arrays sample the scalp at the highest two-dimensional density we know of in the EEG literature. Although this is a significant technical innovation, there is debate about whether increased scalp density is valuable in practice. Some researchers have argued that existing commercial EEG sensor arrays are already dense enough to extract all meaningful information from scalp voltage topographies (Nunez and Srinivasan 2006). The skull and scalp spatially low pass brain potentials and a high number of electrodes may oversample the resulting scalp voltage topography (i.e. exceed the spatial Nyquist rate of the scalp; (Srinivasan *et al* 1998, Nunez and Srinivasan 2006)). However, methods for estimating the scalp spatial Nyquist may be inaccurate because they have typically assumed idealized physical models which may not hold in reality. Additionally, the reconstruction of brain sources may benefit from densities of up to thousands of electrodes (Grover and Venkatesh 2017). Therefore, high-density arrays may have utility for a variety of purposes.

Experimental evidence may be necessary because theoretical analyses disagree about the potential uses of ultra-high-density EEG arrays. Experimental data already support that densities beyond 256 electrodes are practically useful in a variety of EEG subdomains. These include decoding SSVEP (Robinson *et al* 2017), classifying brain states (Petrov *et al* 2014), and recording from neonates (Odabaee *et al* 2013). Additional density could potentially benefit techniques that have already demonstrated improvement with electrode counts up to 96, 128, or 256 electrodes, including localizing and monitoring epilepsy (Lantz *et al* 2003, Nemtsas *et al* 2017) and detecting subcortical EEG sources (Seeber *et al* 2019). The availability of dry, passive, ultra-high-density arrays may help to accelerate discoveries in this area.

### 4.1. Limitations

The exploratory nature of the device fabrication in this study may have resulted in more variability than would be present in bulk fabrication, which could have reduced the similarity between MXtrode signals. Hand-inking the MXtrode arrays likely contributed the most variability to our process. In the future, automated methods like inkjet printing could greatly reduce this variability. Our design did not counterbalance the locations of the electrodes. However, a scalp location effect would not likely lead to our observed results. Electrode spacing, geometry, and potentially the material properties of Ti_3_C_2_T*_x_* MXene are more likely sources of signal variability. Some MXtrode channels were rejected due to high impedance, which may also have been caused by fabrication variability. These rejections led to minor inconsistencies in which MXtrodes were used to form pairs, but the high density of the MXtrode arrays likely minimized the impact of these effects. Our channel rejection strategy occasionally identified contiguous sections of some arrays, which we inferred as likely physical disconnection of the array from the scalp due to inadequate head wrapping. Future application strategies would benefit from methods for generating more uniform (though not more intense) pressure across MXtrode arrays. In addition, the MXtrodes we used had a smaller diameter than the Ag/AgCl electrodes used for comparison, making it challenging to discern whether signal differences were due to geometry or material properties. Future studies could clarify this by comparing similar geometries across different electrode types. Additionally, we recorded from only a limited number of scalp locations. In the future, full-head recordings will be important to confirm the generalizability of our findings. Finally, our analyses of recordings from a site measuring EEG through diverse hair types suggested that the arrays reported in this manuscript recover 1/*f* spectral power distributions and MXtrode timeseries are well-correlated with synchronous Ag/AgCl measurements, which indicate that they are sensitive neural signals across diverse hair types. Adequate skin preparation may be required to record optimal EEG signals from dry MXtrodes, and additional manufactured geometries could be further optimized for adequate scalp contact through the hair and to further minimize impedance.

## 5. Conclusions

We observed that the differences in signal between dry MXtrodes and Ag/AgCl electrodes were mostly similar or smaller than the difference between two Ag/AgCl electrodes at the same distance on the scalp across metrics comparing instantaneous activity, average event-locked signals, amplitude, and spectral properties. Therefore, researchers can use dry MXtrodes to record non-inferior signals to those obtained using gelled Ag/AgCl electrodes for the same research purposes, including through diverse hair types, if adequate skin contact is maintained. The low-profile MXene array used to record EEG in this study requires minimal preparation and no gel, which could significantly speed up and improve the tolerability of basic research applications and the development of new BCI applications. In addition, we showed that MXtrode arrays can record signals independently, without bridging, at a spatial density four times higher than that achievable with gelled electrodes. This high density allowed us to capture more topographic information in the high-frequency (Beta) range than canonical low-density montages. Ultra-high-density montages, such as those made possible by MXtrodes, may enable more accurate source reconstruction and have potential applications in neonatal and epileptic populations. MXtrodes represent a significant advance that may simplify basic EEG research and open new domains for EEG applications.

## Data Availability

The data that support the findings of this study are openly available at the following URL/DOI: https://doi.org/10.6084/m9.figshare.c.6696429.v2.

## CRediT author statement

**Brian Erickson**: Supervision, Conceptualization, Methodology, Software, Data Curation, Writing—Original Draft, Visualization; **Ryan Rich**: Conceptualization, Methodology, Software, Validation, Formal Analysis, Investigation, Data Curation, Writing—Review and Editing, Visualization, Project Admin; **Sneha Shankar**: Methodology, Validation, Investigation, Resources, Data Curation, Writing—Review & Editing; **Brian Kim**: Investigation, Software, Data Curation, Writing—Review & Editing, Visualization; **Nicolette Driscoll**: Methodology, Validation, Investigation, Resources, Data Curation, Writing—Review & Editing; **Georgios Mentzelopoulos**: Investigation, Writing—Review & Editing; **Guadalupe Fernandez-Nuñez**: Investigation; **Flavia Vitale**: Supervision, Conceptualization, Methods, Resources, Writing—Review & Editing Project administration, Funding acquisition; **John Medaglia**: Supervision, Conceptualization, Methods, Resources, Writing—Review & Editing, Project administration, Funding acquisition.
